# Beneficial Effects of Evogliptin, a Novel Dipeptidyl Peptidase 4 Inhibitor, on Adiposity with Increased *Ppargc1a* in White Adipose Tissue in Obese Mice

**DOI:** 10.1371/journal.pone.0144064

**Published:** 2015-12-03

**Authors:** Yu-Na Chae, Tae-Hyoung Kim, Mi-Kyung Kim, Chang-Yell Shin, Il-Hoon Jung, Yong Sung Sohn, Moon-Ho Son

**Affiliations:** Research Institute of Dong-A ST Co., Ltd., Yongin-si, Gyeonggi-do, 446–905, Republic of Korea; Monash University, AUSTRALIA

## Abstract

Although dipeptidyl peptidase 4 (DPP4) is an adipokine known to positively correlate with adiposity, the effects of pharmacological DPP4 inhibition on body composition have not been fully understood. This study was aimed to assess the effects of DPP4 inhibitors on adiposity for the first time in the established obese mice model. The weight loss effects of multiple DPP4 inhibitors were compared after a 4 week treatment in diet-induced obese mice. In addition, a 2 week study was performed to explore and compare the acute effects of evogliptin, a novel DPP4 inhibitor, and exenatide, a glucagon-like peptide-1 (GLP-1) analogue, on whole body composition, energy consumption, various plasma adipokines and gene expression in white adipose tissue (WAT). After the 4 week treatment, weight loss and blood glucose reductions were consistently observed with multiple DPP4 inhibitors. Moreover, after 2-week treatment, evogliptin dose-dependently reduced whole body fat mass while increasing the proportion of smaller adipocytes. However, insulin sensitivity or plasma lipid levels were not significantly altered. In addition to increased active GLP-1 levels by plasma DPP4 inhibition, evogliptin also enhanced basal metabolic rate without reduction in caloric intake, in contrast to exenatide; this finding suggested evogliptin's effects may be mediated by pathways other than via GLP-1. Evogliptin treatment also differentially increased *Ppargc1a* expression, a key metabolic regulator, in WAT, but not in skeletal muscle and brown adipose tissue. The increased expression of the downstream mitochondrial gene, *Cox4i1*, was also suggestive of the potential metabolic alteration in WAT by DPP4 inhibitors. We are the first to demonstrate that pharmacological DPP4 inhibition by evogliptin directly causes fat loss in established obese mice. In contradistinction to exenatide, the fat-loss effect of DPP4 inhibitor is partly attributed to enhanced energy expenditure along with metabolic changes in WAT. These results provide insight into the regulation of energy storage in WAT caused by DPP4 inhibition.

## Introduction

Obesity is closely related to insulin resistance and type 2 diabetes because adipose tissue, which secretes various adipokines with autocrine and paracrine action, is central to the control of energy storage and whole body insulin sensitivity [1]. Recently, dipeptidyl peptidase 4 (DPP4; also known as CD26, EC3.4.14.5) was identified as a novel adipokine potentially linking obesity to metabolic syndrome [2]. DPP4 has gained considerable interest as an antidiabetic target for lowering blood glucose levels; inhibition of DPP4 enzyme activity increases endogenous intact glucagon-like peptide-1 (GLP-1), thereby stimulating insulin secretion which subsequently lowers blood glucose. Therefore, multiple DPP4 inhibitors have been developed for treating type 2 diabetes [3–4]. Although these DPP4 inhibitors have been reportedly neutral with regards to their impact on body weight in humans [3, 5], their effects on body composition have not yet been reported. Animal studies have revealed conflicting results on the effect of DPP4 inhibitors on body weight. In one study, DPP4 inhibitor reduced fat pad weight and adipocyte size in weight gaining mice [6], whereas other studies showed no changes in body weight [7–10]. Meanwhile, there are no reports, to our knowledge, investigating the effects of DPP4 inhibitors on the weight loss and whole body composition in established obese mice.

GLP-1 receptors also exist on the surface of adipose tissue and their activation can cause adipogenesis [11] but enhance lipolysis [12]. GLP-1 analogues have shown to reduce body weight by restricting caloric intake [13–15]. In contrast to GLP-1 analogues and genetic deficiency of DPP4 [16], DPP4 inhibitors do not affect food consumption [8, 17], suggesting its effects in energy homeostasis may be governed by different mechanisms of action. Increased GLP-1 action by pharmacological DPP4 inhibition partly accounts for reduced adiposity in weight-gaining mice through improving lipid metabolism [18]. Due to the limited secretion of GLP-1, plasma levels of endogenous GLP-1 enhanced by inhibiting DPP4 are also limited, yet a much higher plasma level of exenatide, a GLP-1 analogue, is needed for body fat loss than for glucose control. Thus it has been unclear whether the effect of DPP4 inhibitors on reducing adiposity can be fully explained by increased circulating GLP-1 levels.

There is very limited information about the direct effects of DPP4 inhibitors on the adipokine action of DPP4 [2], independent of GLP-1 action. DPP4 is highly expressed in kidneys and also exists in various types of adipose tissue such as visceral and to a lesser extent, subcutaneous fat tissue [2, 18]. Adipocytic DPP4 expression increases according to adipocyte differentiation [19] with a positive correlation between plasma DPP4 and body weight [20–21]. This evidence provides a potential strong linkage between DPP4 and adiposity. Although DPP4-deficient mice with at least 6-fold higher ambient plasma GLP-1 levels had less body weight and fat mass due to the increased energy expenditure and reduced food intake when fed on a high-fat diet [16], GLP-1 dependent or independent effects on adiposity have not been dissected yet. Fukuda and colleagues thereafter hypothesized that energy dissipation in muscle can be the potential underlying mechanism of reduced weight gain by DPP4 inhibition after a 10-week long-term treatment [22]. However, because observed effects on fat tissue in long-term studies can be confounded by multiple factors by way of both direct and indirect actions, our understanding of the direct effects of DPP4 inhibitors on fat tissue has remained nebulous.

Herein we investigated the direct effects of our novel DPP4 inhibitor, evogliptin [17, 23–24], on body composition and adiposity in an established obese mice model. Additionally, the effects of evogliptin were compared with those of exenatide, a GLP-1 analogue.

## Materials and Methods

### Materials

Evogliptin ((R)-4-[(R)-3-amino-4-(2, 4, 5-trifluorophenyl)butanoyl]-3-(t-butoxymethyl) piperazin-2-one, purity > 97.0%) L-tartrate salt was synthesized in house [24]. Sitagliptin phosphate and vildagliptin were purchased from Jiacheng-Chem (Hangzhou, China). Saxagliptin hydrate and linagliptin were products of APIchem (Shanghai, China) and Nanjing Chemlin (Nanjing, China), respectively. Gly-Pro-7-amido-4-methylcoumarin (Gly-Pro-AMC) as a substrate for the DPP4 enzyme and exenatide were obtained from Merck (Frankfurt, Germany) and American Peptide (Sunnyvale, CA), respectively. All other chemicals unless otherwise specified, were purchased from Sigma-Aldrich (St. Louis, MO).

### Ethics statement

All experiments were performed in compliance with Korean legislation under the Laboratory Animal Act 2009 and were approved by the Institutional Animal Care and Use Committee of Dong-A ST Research Institute. Male C57BL/6J mice at age of 6 weeks were obtained from Dae Han Biolink (Eumseong, Korea). Mice were maintained at 23 ± 2°C on a 12:12-h light-dark cycle (lights on 0700 –1900h) and housed 2 animals per cage. Mice were allowed free access to food and water.

### High-fat diet-induced obese (HF-DIO) mice studies

Seven-week-old mice were fed a high-fat diet (60% fat and 20% carbohydrates [kcal 100 g^-1^], D12492, Research Diets, New Brunswick, NJ) *ad libitum*. After 14 weeks on a high-fat diet, the mice were housed two animals per cage and were individually acclimated in metabolic cages for 1 or 2 weeks. As a lean control, age-matched male C57BL/6J mice were kept on a normal chow diet. Each repeated dosing study is summarized in Table 1.

**Table 1:** Study design.

	Duration of treatment	Dosing route/interval	Drug	Daily dose		Outcome measures
**Study 1**						
	4 weeks	Twice daily by oral gavage	Evogliptin		60 mg kg^-1^	body weight, plasma GLP-1,
			Sitagliptin		300 mg kg^-1^	plasma insulin, blood glucose
			Vildagliptin		200 mg kg^-1^	
			Saxagliptin		60 mg kg^-1^	
			Linagliptin		10 mg kg^-1^	
**Study 2**						
	2 weeks	Once daily s.c. inj.	Exenatide		30 μg kg^-1^	body composition, plasma DPP4 activity,
		Drug-diet mixture	Evogliptin	0.027%	20 mg kg^-1^	food consumption,
				0.081%	60 mg kg^-1^	plasma TG, histology of WAT
				0.27%	200 mg kg^-1^	
**Study 3**						
	2 weeks	Once daily s.c. inj.	Exenatide		30 μg kg^-1^	body composition, energy expenditure,
		Drug-diet mixture	Evogliptin	0.081%	60 mg kg^-1^	plasma insulin, adipokines,
						gene expression, rectal temperature

#### Study 1

HF-DIO mice were allocated to each group (8 animals/group) according to body weight and blood glucose levels. We selected preliminary doses of evogliptin, sitagliptin, vildagliptin, saxagliptin, and linagliptin in HF-DIO mice based on the doses used elsewhere [17, 25–27, 10]. To confirm their pharmacodynamic effects, plasma DPP4 inhibition was assessed 12 h after single oral administration of each drug. Due to the observed short half lives of sitagliptin and evogliptin in mice (unpublished data), all the drugs were administered twice daily by oral gavage and given for 4 weeks using 0.5% (w/v) methylcellulose as a vehicle. Body weight and diet consumption were measured weekly. Before the last administration, fed blood glucose levels were measured from the tail vein blood using a glucometer (AccuChek Active, Roche Diagnostics, Mannheim, Germany). Twelve hours after the last administration, mice were euthanized in the non-fasted state and fed GLP-1 and insulin levels were determined. Blood for determining active GLP-1 was collected in heparinized tubes with sitagliptin (final 1 mM) to minimize the degradation of GLP-1.

#### Study 2

HF-DIO mice were allocated to each group (8 animals/group) according to body weight and whole body fat mass. To determine the acute effects on body composition, treatment period was shortened to 2 weeks. In this study, evogliptin at three doses targeting 20, 60, and 200 mg kg^-1^ day^-1^ for determining dose response and exenatide at the moderate dose were compared. Considering the half-life (2–3 h) of exenatide in mice and the minimum effective dose of 1 μg kg^-1^ based on oral glucose tolerance test in mice after single subcutaneous injection of exenatide (unpublished data), we designated 30 μg kg^-1^ day^-1^ as the moderate dose. To reduce the stress caused by animal handling, evogliptin was given to mice as a drug-diet admixture (0.027, 0.081, or 0.27% (w/w)) and exenatide was subcutaneously given to mice once daily. Mice which did not receive exenatide received subcutaneous injections of saline. Control and exenatide-treated mice were fed on a repelleted diet without evogliptin. Diet consumption and body weight were measured daily for 2 weeks. Body composition was measured before and after treatment. After euthanasia in the non-fasted state, plasma was obtained for determining plasma DPP4 activity and triglycerides levels. Because epididymal fat mass was highly correlated with whole body fat mass (unpublished data; Pearson’s correlation coefficient = 0.949, *P*<0.0001, n = 60), epididymal white adipose fat pad (WAT) was isolated and immediately frozen for histology.

#### Study 3

HF-DIO mice were allocated to each group (8 animals/group) according to body weight and whole body fat mass. To explore the underlying mechanisms of fat loss, HF-DIO mice received either daily subcutaneous injections of 30 μg kg^-1^ day^-1^ of exenatide or saline with a high-fat diet with/without 0.081% (w/w) evogliptin. Two weeks after treatment, rectal temperature was determined and indirect calorimetry was performed. Body composition was measured before and after treatment. After euthanasia in the non-fasted state, plasma was obtained for determining plasma insulin and adipokine panel. Given that soleus muscle mass directly reflects metabolic change [28] and interscapular fat is representative of brown adipose tissue (BAT) and widely used to investigate the metabolic effects on BAT [29–30], soleus muscle, interscapular BAT, and epididymal WAT were isolated and immediately frozen for evaluation of gene expression.

### Body composition analysis

Body composition was measured under conscious state using a Minispec LF90II NMR spectrometer (Bruker Optics, Ettlingen, Germany) before and after treatment. Lean body mass was obtained by subtracting fat mass from body weight.

### Histological evaluation of adipose tissue

Epididymal adipose tissues were removed, prepared for 4 μm-thick microtome sections, and then mounted for hematoxylin-eosin staining. Images were obtained using an Axioskop2 Plus microscope (Carl Zeiss, Gottingen, Germany), and adipocyte area was measured using ImageJ (http://rsbweb.nih.gov/ij). After processing background of images, the cellular boundary line was automatically detected and manually compared to the original image. Then the area of each cell in the same area was automatically determined except for cells appearing partly at the edge of image frame. Three sections per animal were analyzed.

### Determination of plasma parameters

Plasma DPP4 activity was measured using Gly-Pro-AMC as previously described [15]. Plasma triglycerides were determined using an automatic analyzer (Reflotron^®^ Plus System, Roche, Mannheim, Germany). Plasma insulin was measured using a Milliplex Mouse Adipokine Panel assay service (Millipore, St. Charles, MO). Plasma active GLP-1 and leptin levels were measured using ELISA kits from LINCO Research (St. Charles, MO). An ELISA kit for high molecular weight adiponectin from Shibayaki (Shibukawa, Japan) was used for detecting biologically active form of adiponectin.

### Indirect calorimetry

After the 2-week treatment, mice were transferred to a new cage to evaluate metabolic rate and accommodated under free access to food and water. Data were obtained for 21 h using a computer-controlled open-circuit indirect calorimetry system (Oxylet system; Panlab Harvard Apparatus, Barcelona, Spain) with airflow of 0.35~0.37 l min^-1^, and metabolic parameters such as oxygen consumption, carbon dioxide production and mean energy expenditure were automatically assessed according to manufacturer’s instructions. Respiratory quotient (Rq) was computed by dividing carbon dioxide production by oxygen consumption.

### RT-PCR

Information of primers and probes used is listed in S1 Table. For mouse peroxisome proliferator-activated receptor gamma coactivator 1-alpha (*Ppargc1a*) and hypoxia inducible factor 1-alpha (*Hif1a*), SYBR Green I Master Mix was used in a LightCycler 480 (Roche Applied Science, Indianapolis, IN). TaqMan probe method was used for mouse uncoupling protein 1 (*Ucp1*) and cytochrome c oxidase subunit 4 isoform 1 (*Cox4i1*). Data analysis was performed using standard curve method. Relative gene expression was presented after normalization by mouse ribosomal protein S3 (*Rps3*) expression (when fold difference = 2^-ΔCt^, an average fold change of < 2 and a maximal variability of < 6-fold between samples) which was validated as described elsewhere [31].

### Statistical analysis

All data was expressed as means ± SE. Except for ANCOVA, statistical analyses were performed using SigmaStat^®^ 2.0 (SPSS, Chicago, IL). Comparisons with baseline values and comparisons between two groups were analyzed using Student’s paired *t*-test and Student’s *t*-test, respectively. For comparisons of data involving more than three groups at the same time point, one-way analysis of variance (ANOVA) was used. For comparisons of data at different time points from the same animals among more than three groups, we used repeated measures (RM) two-way ANOVA. When ANOVA indicated a significant difference, the differences were evaluated further using Dunnett’s multiple comparison test. The adipocyte size distribution was analyzed using the Mann—Whitney rank sum test between two groups due to data skewness. *P* values < 0.05 were considered significant. One-way analysis of covariance (ANCOVA) was performed using SigmaPlot^®^ 13.0 (Systat Software, Inc., San José, CA).

## Results

### Weight-loss effect of DPP4 inhibitors in HF-DIO mice

**Fig 1:**
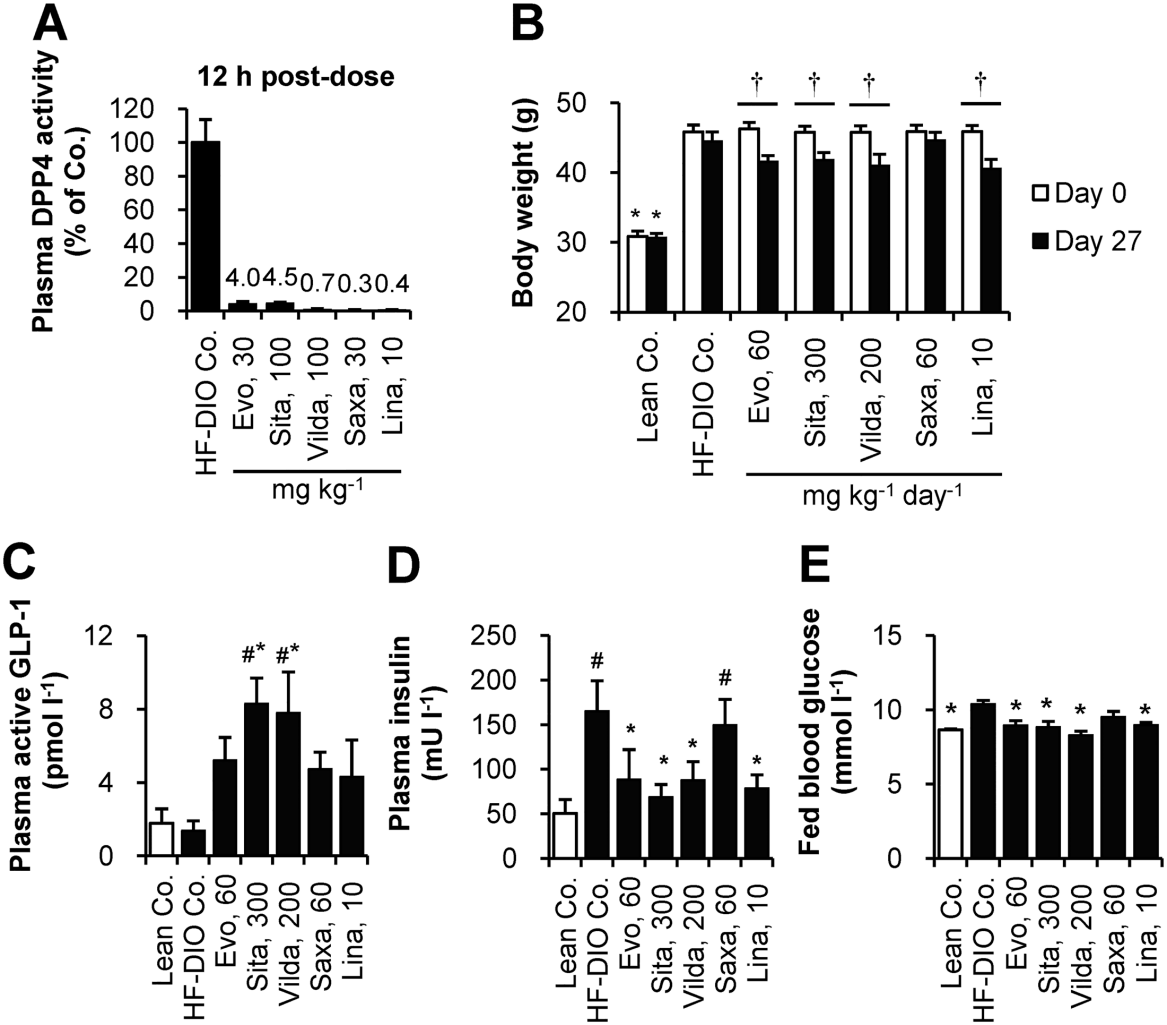
Body weight-loss effect of DPP4 inhibitors in established obese mice after 4-week treatment. (A) To select each dosage for twice daily administration, plasma DPP4 inhibition was assessed 12 h after a single oral administration in HF-DIO mice. (B-E) After 4 week-administration of evogliptin, sitagliptin, vildagliptin, saxagliptin, or linagliptin twice daily by oral gavage in HF-DIO mice, (B) body weight, (C) fed plasma levels of active GLP-1, (D) insulin, and (E) fed blood glucose levels were determined (n = 5/group). #, *P* < 0.05 vs. lean control; *, *P* < 0.05 vs. HF-DIO control by one-way ANOVA; †, *P* < 0.05 vs. the baseline by RM two-way ANOVA.

For dose selection, plasma DPP4 activity was assessed 12 h after single oral administration of each drug at a preselected dose in HF-DIO mice. All the DPP4 inhibitors showed over 90% inhibition of plasma DPP4 activity at the doses tested (Fig 1A). Given that DPP4 inhibition over 80% was considered clinically meaningful [32], those tested doses were selected for the next study phase except for sitagliptin whose dose was increased from 100 mg kg^-1^ to 150 mg kg^-1^ twice daily for full efficacy based on a reported target daily dose of 280 mg kg^-1^ [25].

After four weeks of treatment to HF-DIO mice, body weight-loss effects were observed and statistically equivalent among four DPP4 inhibitors except for saxagliptin (Fig 1B). Cumulative diet consumption did not differ among treatment groups (data not shown). As expected, plasma DPP4 inhibition caused an increase of active GLP-1 levels, with the highest levels observed in sitagliptin or vildagliptin treatment groups at the doses tested (Fig 1C). However, basal insulin levels similarly declined in evogliptin, sitagliptin, vildagliptin and linagliptin-treated mice, indicative of improved insulin sensitivity. These results supported the significant reduction in blood glucose levels by four DPP4 inhibitors (Fig 1E). The changes in basal insulin and blood glucose were concordant with body weight loss across all drugs except saxagliptin whose effects on weight were not significant despite similar plasma DPP4 inhibition and increase of active GLP-1 levels.

### Acute effect of evogliptin on whole body composition in HF-DIO mice

Next, we compared treatment response to evogliptin and exenatide to examine whether the observed weight loss was directly due to DPP4 inhibition. To confirm a dose response, evogliptin, as a drug-diet mixture, was given to mice at three doses including lower and higher dosages than the dose of evogliptin (60 mg kg^-1^ day^-1^) which was tested in the 4-week study. Mean administered doses were 21.0±1.3, 62.5±2.0, and 202.7±6.2 mg kg^-1^ day^-1^ for 0.027, 0.081, and 0.27% (w/w), respectively.

**Fig 2:**
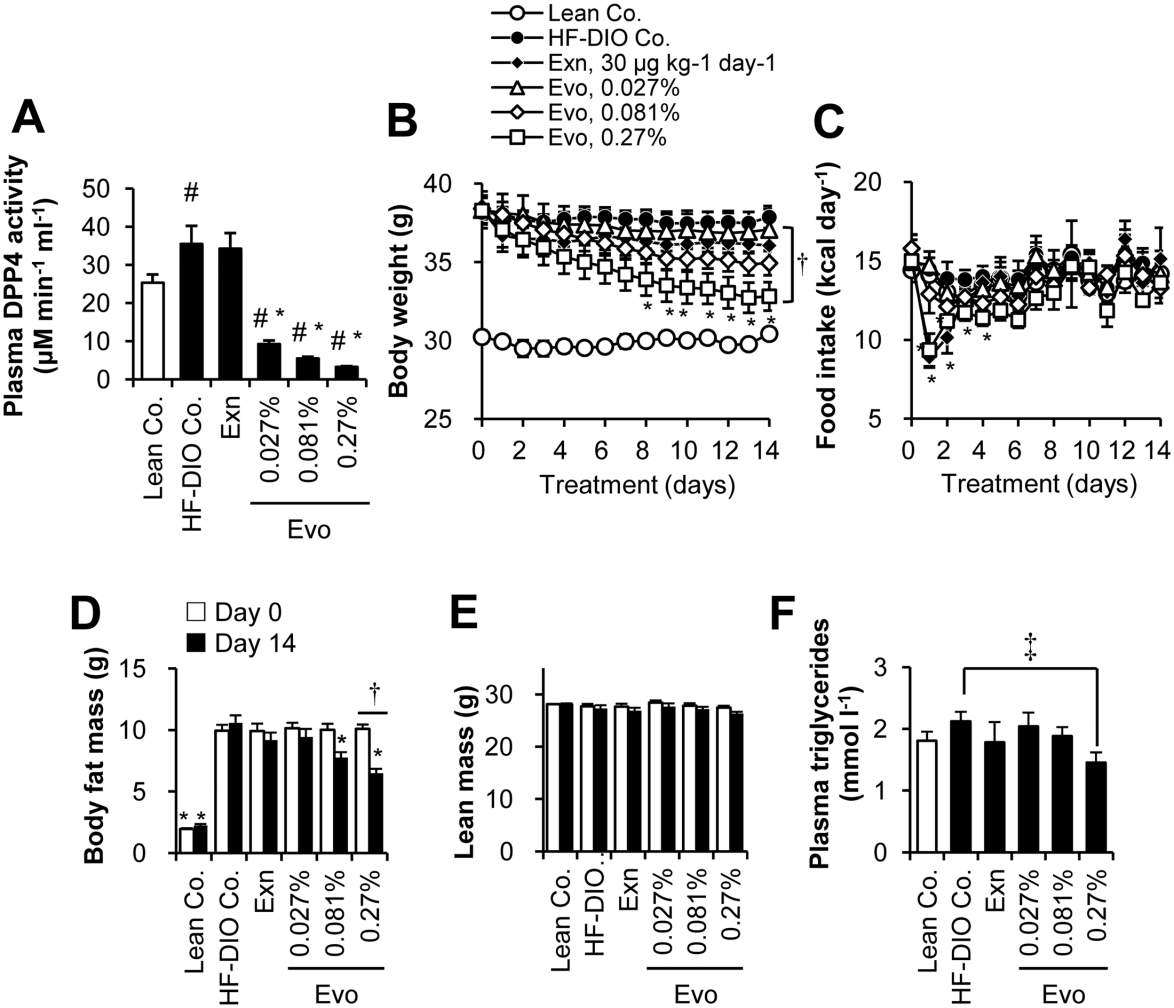
Two-week treatment of evogliptin decreased whole body fat mass in obese mice. After HF-DIO mice were treated with exenatide (30 μg kg^-1^, once daily, s.c.) or evogliptin at 0.027%, 0.081%, or 0.27% (w/w) for 2 weeks, (A) plasma DPP4 activity, (B) body weight, (C) energy intake, (D) whole body fat mass, (E) lean body mass, and (F) plasma triglycerides levels were determined (n = 8/group). #, *P* < 0.05 vs. lean control and *, *P* < 0.05 vs. HF-DIO control by one-way ANOVA; †, *P* < 0.05 vs. the baseline by RM two-way ANOVA; ‡, *P* < 0.05 vs. HF-DIO control by Student’s *t*-test.

After 2-week treatment, evogliptin caused dose-dependent inhibition of plasma DPP4 activity (74.0%, 84.5%, and 90.7% at doses of 0.027%, 0.081%, and 0.27%, respectively)(Fig 2A). Evogliptin treatment at 0.27% continuously decreased body weight with a significant reduction of 13.3% vs. HF-DIO control (Fig 2B; *P* < 0.05 by RM two-way ANOVA). However, evogliptin at 0.081% and exenatide treatments did not show significant weight reductions (-7.74% and -4.77% vs. HF-DIO control, respectively). Furthermore, a return to a lean mouse phenotype was not observed with any of the treatments.

Daily energy intake acutely decreased on day 1 by 0.27% evogliptin and exenatide alone and then recovered 3–5 days afterwards (Fig 2C). However the pattern of energy intake of the same animals was unchanged and neither was cumulative diet consumption (data not shown). The body weight in 0.081% and 0.27% evogliptin-treated mice continuously declined throughout the treatment period regardless of energy intake; meanwhile, weight-loss by exenatide treatment was maintained after a rapid drop on the second day of administration, implying the effects of either drug were driven by different pharmacodynamics.

As for whole body fat mass, evogliptin treatment dose-dependently lowered fat mass with the greatest reduction achieved at the dose of 0.27% (-38.5% vs. HF-DIO Co., *P* < 0.05 by RM two-way ANOVA)(Fig 2D, S2 Table). Although there was no statistically significant difference in fat-loss trend (*P >* 0.05 by RM two-way ANOVA), evogliptin 0.081% treatment exhibited a greater reduction in fat mass than exenatide (-26.6% vs -13.0%, respectively) when compared to HF-DIO control after 2 week-treatment (*P* = 0.002 and *P* = 0.147 by one-way ANOVA, respectively). Lean body mass was not significantly altered by any treatments (*P* = 0.389)(Fig 2E). Plasma triglycerides levels were lower only at 0.27% evogliptin-treated mice relative to HF-DIO Co. (*P* = 0.012 by Student’s t-test)(Fig 2F). These results indicated an acute fat-loss effect due to DPP4 inhibition.

### Acute effects on adiposity in HF-DIO mice

**Fig 3:**
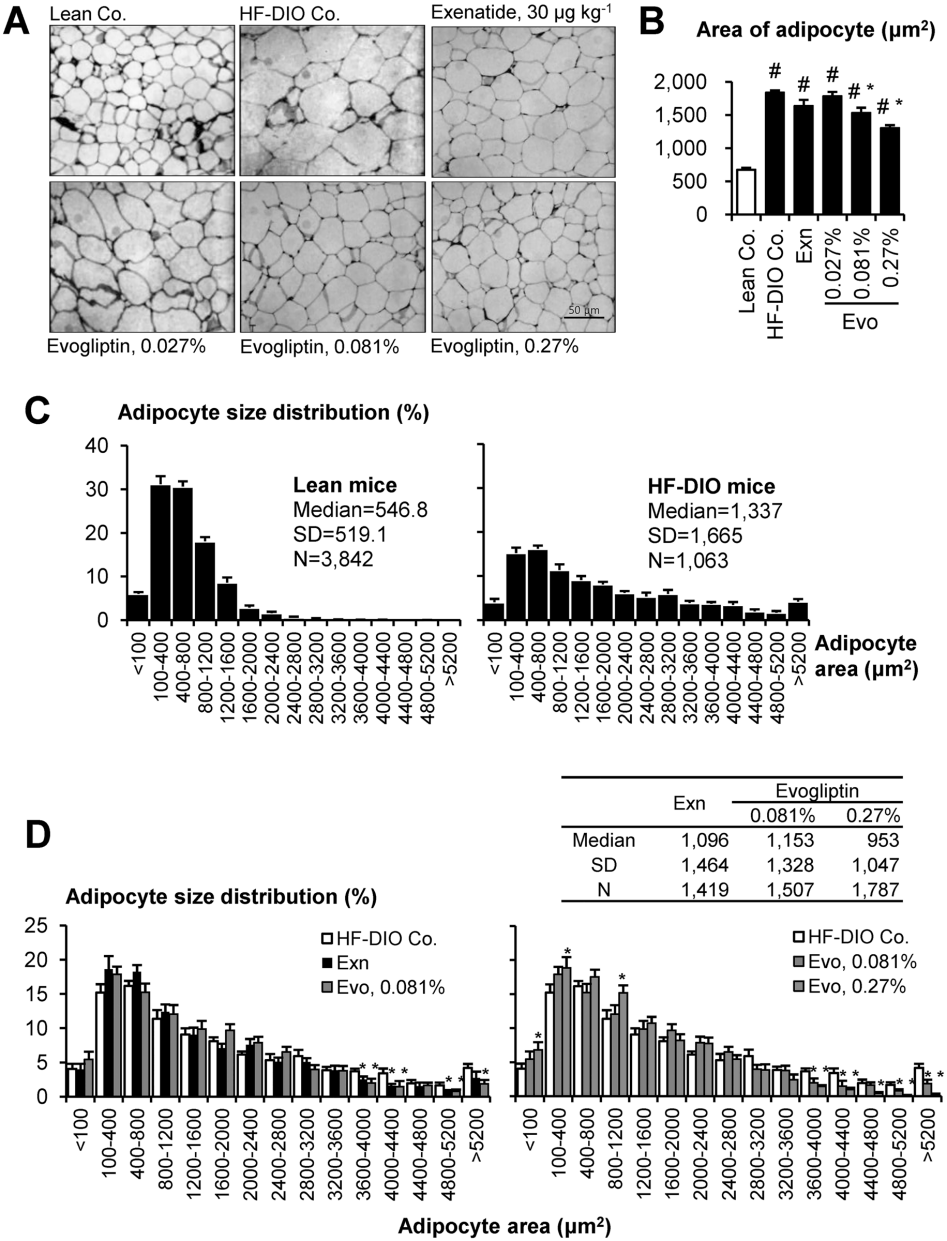
Evogliptin treatment reduced adipocyte size and distribution. HF-DIO mice were treated with exenatide (30 μg kg^-1^, once daily, s.c.) or evogliptin at 0.027%, 0.081%, or 0.27% (w/w) for 2 weeks. The effects on adipocytes from epididymal fat tissues were histologically determined (three sections/animals, eight animals/group). (A) Representative images (original magnification, ×200), (B) mean adipocyte areas and (C-D) percentile distribution of adipocyte size were presented. #, *P* < 0.05 vs. lean control and *, *P* < 0.05 vs. HF-DIO control by one-way ANOVA.

We histologically analyzed adipocyte size distribution in epididymal fat section. HF-DIO mice typically exhibited prominent adipocyte hypertrophy (Fig 3A and 3B) and the size distribution was significantly right-shifted in HF-DIO mice compared to that in lean mice (*P* < 0.05, Mann—Whitney rank sum test), indicating an increased proportion of large adipocytes (Fig 3C). Evogliptin treatment dose-dependently reduced mean adipocyte size and left-shifted its size distribution (Fig 3D). It was a noteworthy finding that the percentage of large size adipocytes > 3 600 μm^2^ decreased significantly in a dose-dependent manner. Intriguingly, when the 2-week evogliptin treatment significantly reduced whole body fat mass at 0.081%, mean adipocyte size and the median value of adipocyte size distribution was significantly improved compared to that of the HF-DIO control (*P* < 0.05, Mann—Whitney rank sum test). The adipocyte size distribution was comparable between exenatide- and 0.081% evogliptin-treated groups. Compared to exenatide, evogliptin treatment at 0.27% showed a similar reduction in initial diet consumption but was superior in amelioration of adiposity to exenatide (*P* < 0.05, Mann—Whitney rank sum test), which also suggested an additional fat-loss inducing mechanism of action by evogliptin treatment.

### Evogliptin increased energy expenditure unlike exenatide

The effects on glucose metabolism were compared at the end of 2-week treatment. Both evogliptin at 0.081% and exenatide did not significantly improve the insulin sensitivity (11–13%, *P* > 0.09 by One-way ANOVA) (S1A and S1B Fig). Notably, while fat-loss effects by both treatments were comparable, reduction of 6 h-fasted blood glucose was doubled in exenatide-treated mice compared to 0.081% evogliptin (-29.5% vs. -11.9%, respectively; *P* < 0.05)(S1C Fig). Endogenous active GLP-1 levels were not altered by exenatide treatment (S1D Fig).

**Fig 4:**
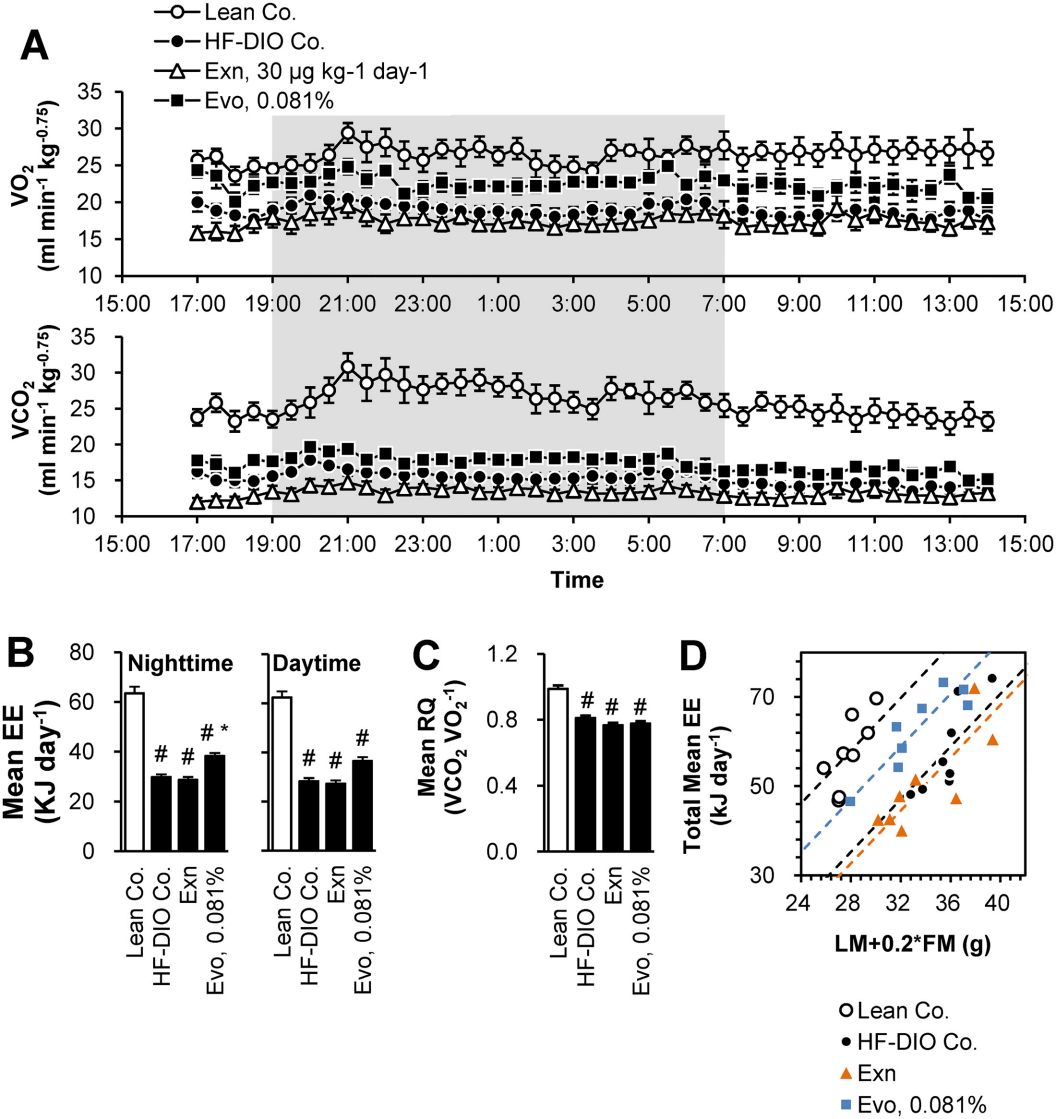
Evogliptin increased energy expenditure unlike exenatide. HF-DIO mice were treated with exenatide (30 μg kg^-1^, once daily, s.c.) or evogliptin at 0.081% (w/w) for 2 weeks. Metabolic parameters were measured using an indirect calorimetry system; (A) Oxygen consumption (VO_2_) and carbon dioxide production (VCO_2_), (B) mean energy expenditure (EE), (C) respiratory quotient (Rq), and (D) a plot of individual data of total mean EE vs. (lean mass + 0.2 × fat mass) as a covariate (n = 8/group). #, *P* < 0.05 vs. lean control and *, *P* < 0.05 vs. HF-DIO control by one-way ANOVA.

Because restricted energy intake was not a cause of fat-loss by 0.081% evogliptin, we examined the acute effects on energy expenditure. In Study 3, when the changes in body composition of Study 2 were recapitulated (S2 Fig), 0.081% evogliptin treatment enhanced oxygen consumption, carbon dioxide production (Fig 4A) and energy expenditure especially at nighttime (Fig 4B). Meanwhile, exenatide treatment had no effect on energy expenditure. Neither evogliptin nor exenatide treatment altered the respiratory quotient (Fig 4C). Because energy expenditure can be altered by both lean body mass and fat mass, mean total energy expenditure was examined according to body composition (S3 Fig). When the effect of treatment on energy expenditure was comprehensively analyzed by ANCOVA using [lean mass+0.2×fat mass] as a covariate as previously described [33], the equal slope assumption passed indicating no interactions between each treatment and covariates. Interestingly, adjusted means of energy expenditure were greater in evogliptin-treated mice than HF-DIO control or exenatide-treated mice. Moreover, in this equal slope model (R^2^ = 0.717, adjusted R^2^ = 0.675), the y-intercept of evogliptin treatment was greater than those of HF-DIO control and exenatide (-35.8 vs. -47.6 and -50.2, respectively; *P* < 0.001, ANCOVA) (Fig 4D), indicative of higher energy expenditure in evogliptin treated-mice. However, there were no differences in core body temperature (S4 Fig). This result suggested that part of evogliptin-induced fat loss may be attributed to the enhanced metabolic rate which may not be mediated by increased active GLP-1.

### Effects on plasma parameters

**Fig 5:**
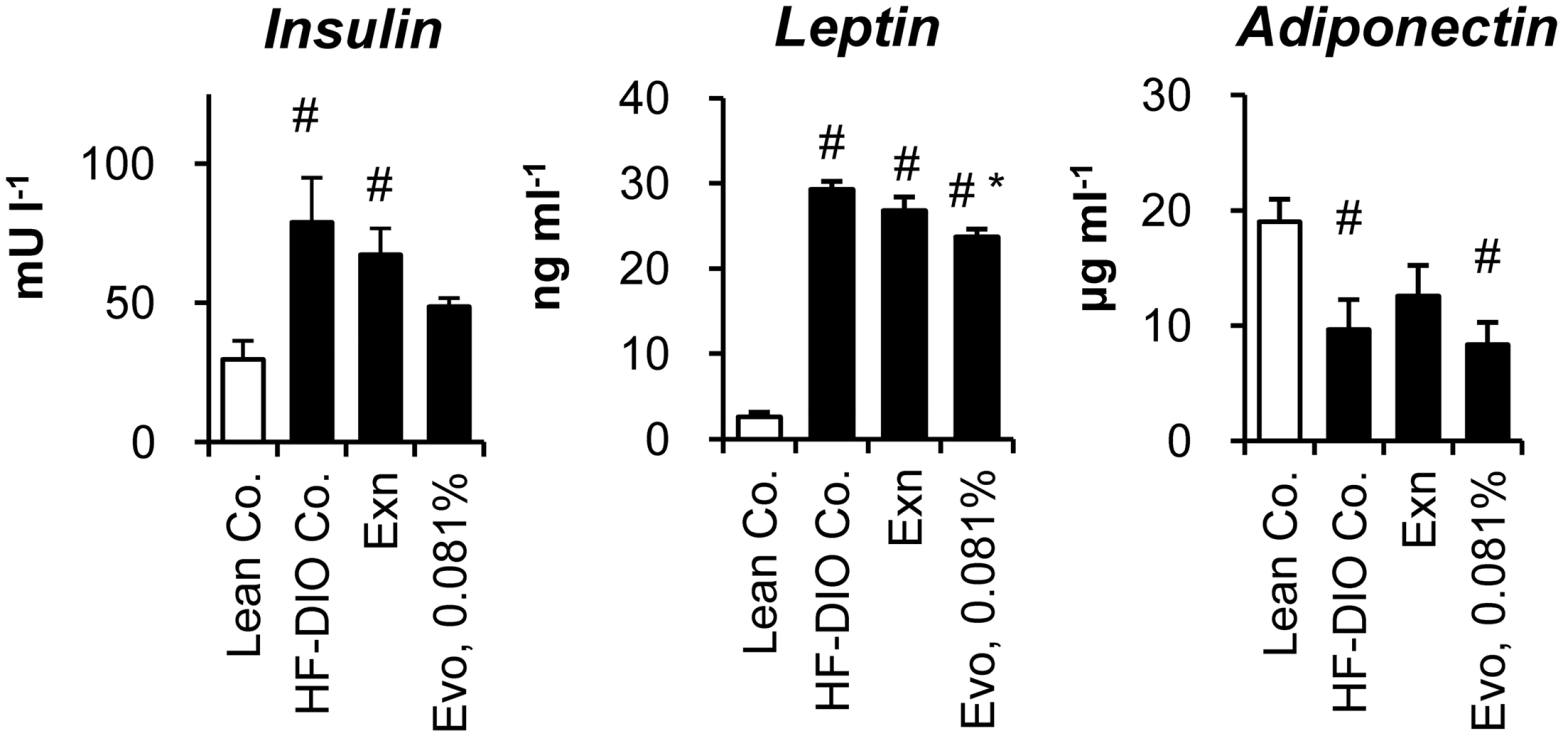
Effects on plasma insulin and adipokines. After 2-week treatment of exenatide (30 μg kg^-1^, once daily, s.c.) or evogliptin at 0.081% (w/w) in HF-DIO mice, fed plasma concentrations of (A) insulin, (B) leptin and (D) high molecular weight (HMW) adiponectin were assessed (n = 8/group). #, *P* < 0.05 vs. lean control and *, *P* < 0.05 vs. HF-DIO control by one-way ANOVA.

Adipose tissue is an endocrine organ secreting various cytokines that regulate whole body metabolism, as well as a highly active metabolic organ for storing surplus energy. Therefore, we evaluated the effects of each treatment on the plasma levels of insulin and five cytokines. Increased plasma levels of insulin and leptin denoted insulin resistance and leptin resistance in HF-DIO mice (Fig 5). Both exenatide and 0.081% evogliptin alone did not significantly improve hyperinsulinemia in HF-DIO mice (*P* = 0.535 and *P* = 0.071 by One-way ANOVA, respectively), which corroborated the results of the insulin tolerance test. Meanwhile, only 0.081% evogliptin treatment led to a significant reduction of plasma leptin. The physiologically active form of adiponectin stayed lower in obese mice than lean mice, while neither exenatide (*P* = 0.690) nor 0.081% evogliptin altered adiponectin levels.

Plasma levels of plasminogen activator inhibitor-1, another adipokine, and C-reactive protein, as an inflammatory indicator, were higher in obese mice than lean mice, yet these levels were not significantly altered by any treatments (S5 Fig). Plasma levels of tumor necrosis factor α and monocyte chemoattractant protein-1 simultaneously analyzed using a Milliplex kit were under the minimum detection limit of 2.5 pg ml^-1^ and 7.8 pg ml^-1^, respectively (data not shown).

Taken together, these results suggested that reduced adiposity after 2-week treatment of evogliptin was not attributed to alteration of adipokines levels or inflammation.

### Evogliptin increased *Ppargc1a* gene expression in WAT

Increased energy expenditure can be explained by enhanced metabolic rate in skeletal muscle [22] or adipose tissues [34]. Given that PGC1α (*Ppargc1a*) is a key regulator involved in lipid metabolism in various peripheral tissues including muscle [35–36] and adipocyte [37–38], we presumed that evogliptin regulates PGC1α expression in its target organ.

**Fig 6:**
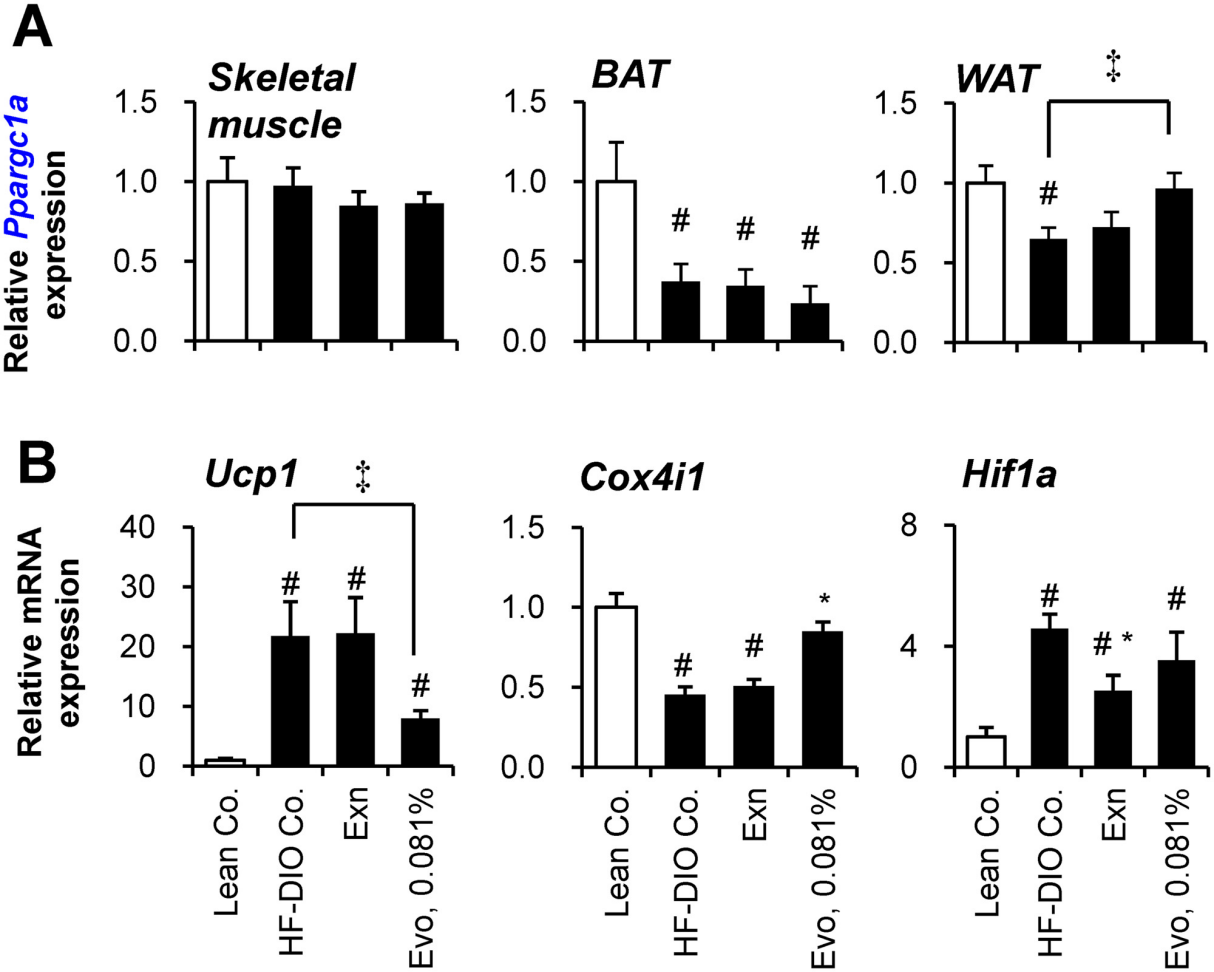
Effect of evogliptin treatment on the gene expression in muscle and adipose tissues. After 2–week administration of exenatide (30 μg kg^-1^, once daily, s.c.) or evogliptin at 0.081% (w/w) in HF-DIO mice, (A) PGC-1α (*Ppargc1a*) gene expression in soleus muscle and two types of fat tissues (interscapular fat and epididymal fat for brown adipose tissue and white adipose tissue, respectively), and gene expression levels of (B) UCP1 (*Ucp1*), (C) COXIV (*Cox4i1*) and HIF-1α (*Hif1a*) were assessed in white adipose tissue by realtime qPCR method (n = 8/group). #, *P* < 0.05 vs. lean control and *, *P* < 0.05 vs. HF-DIO control by one-way ANOVA; ‡, *P* < 0.05 by Student’s t-test.

To determine the target organ of evogliptin, we evaluated the gene expression levels of *Ppargc1a* in skeletal muscle, BAT and WAT with regard to energy homeostasis. *Ppargc1a* expression in skeletal muscle was not altered by a high-fat diet or any treatments (Fig 6A). In BAT, *Ppargc1a* expression was significantly decreased by a high-fat diet, but not affected by any treatments. However, in WAT, evogliptin treatment significantly restored *Ppargc1a* gene expression which was normally suppressed by feeding a high-fat diet, suggesting evogliptin’s influence on metabolic changes in WAT.

Given that PGC1α stimulates thermogenesis via inducing UCP1 expression, gene expression of UCP1 (*Ucp1*) was first examined. Unexpectedly, evogliptin treatment reduced gene expression of *Ucp1* in WAT (Fig 6B), suggesting an alternative pathway may be responsible. Because PGC1α enhances metabolism through up-regulating mitochondrial biogenesis in adipose tissue, the effect of evogliptin on the expression levels of a putative mitochondrial gene, *Cox4i1*, was also examined. High-fat feeding reduced mitochondrial *Cox4i1* gene expression, but evogliptin treatment, unlike exenatide, restored gene expression of *Cox4i1*. Conversely, exenatide, unlike evogliptin, reduced *Hif1a* gene expression enhanced by a high-fat diet. Evogliptin showed a differential reduction in adipocyte gene expression of *Pparg2* contrary to *Srebp1c* and *Dpp4* (S6 Fig). Given that PPARγ2 is a typical adipogenic transcription factor, we examined whether evogliptin directly inhibited the differentiation of human adipocytes. In this assay, evogliptin neither induced adipogenesis per se nor antagonized the adipocyte differentiation induced by rosiglitazone, a typical PPARγ agonist, along with no direct activation of human and mouse PPARα/γ (S6 Fig).

These results indicated that, compared to exenatide, 0.081% evogliptin treatment differentially influenced the lipid metabolism in WAT which may serve the potential target for evogliptin.

## Discussion

Although various gliptins are known to be neutral on body weight in type 2 diabetic patients, the effect on body fat mass has not been fully elucidated in humans and animals yet. To the best of our knowledge, this is the first report on the body weight-loss effect through pharmacologically inhibiting DPP4 activity in established obese mice. We also demonstrated that our novel DPP4 inhibitor, evogliptin, in contrast to exenatide, directly reduced whole body fat mass by enhanced energy expenditure at a clinically relevant dose achieving over 80% plasma DPP4 inhibition for 24 hours. Our findings proposed the possibility that the target organ of evogliptin treatment may be WAT.

An imbalance between energy intake and expenditure causes weight gain or loss. Exenatide produced an anorexic effect at three- to ten-fold higher doses than that for the glucose-lowering effect [6, 39]. On the other hand, the maximum increase in endogenous intact GLP-1 levels enhanced through inhibiting DPP4 activity was limited due to restricted secretion. While chronic treatment with sitagliptin led to peak active GLP-1 levels of around 50 pM after a meal challenge which rapidly declined within 2–3 h in HF-DIO mice [40], evogliptin 0.081% led to mean ambient GLP-1 levels of 10.3±1.01 pM (3.7 fold greater compared to HF-DIO control). This evidence supports the notion that the endogenous GLP-1 levels increased by DPP4 inhibition are insufficient to produce the anorexic effect of GLP-1 signaling.

In this study, the glucose-lowering effect of exenatide at 30 μg kg^-1^ was superior to 0.081% evogliptin, suggesting that the fat-loss effect of evogliptin may not be a result of improvement in glucose metabolism. Among adipokines, 2-week evogliptin treatment significantly lowered only plasma leptin levels. Leptin acts as a negative regulator of energy intake on hypothalamus [41] and plasma leptin levels were lowered by 0.081% evogliptin at the end of treatment. Furthermore, evogliptin at 0.081% did not alter daily diet consumption, but enhanced energy expenditure. These results cannot be fully explained by the improvement of leptin resistance [42], although we did not determine levels of leptin receptors. Thus we concluded that reduction in plasma leptin may be a result of reduced adiposity rather than direct causality with DPP4 inhibition.

We suggested that one possible mechanism in evogliptin-induced fat loss is increased energy dissipation. While it is clear that exenatide decreases food intake by increasing satiety and anorexic effect, the effect of exenatide on energy expenditure was controversial. It was reported that exenatide reduced physical activity, oxygen consumption, and energy expenditure in rodents [43–44]. While some clinical researchers have suggested the possibility of increased energy expenditure [45–47], a recent clinical study, which investigated energy expenditure in obese subjects after exenatide administration, disproved this theory [48]. Goldsmith et al. reported that sitagliptin induced energy expenditure after 10 week-treatment in diabetic mice, which was partially sustained even in GLP-1 receptor-deficient mice [49]. Therefore, this data supports the notion that increased energy expenditure by evogliptin may be partly GLP-1 independent.

In humans, vildagliptin tended to increase energy expenditure and augmented lipid mobilization and fatty acid oxidation after 7-day treatment, suggesting a potential contribution of GLP-1-mediated activation of the sympathetic nervous system [50]. Although several studies reported that DPP4 inhibitors prevented weight gain [6, 18, 22, 51–52], the underlying mechanisms remained not fully understood. Teneligliptin also was reported to increased energy expenditure in weight-gaining mice [22]. The proposed mechanism therein was an increase of energy dissipation in skeletal muscle. Enhanced energy expenditure can be explained by increased metabolic rate and/or thermogenesis in adipose tissues and skeletal muscle [36, 53]. Herein evogliptin treatment did not induce thermogenesis but increased metabolic rate. We discovered that the key metabolic factor was altered in WAT along with alteration of its downstream target gene, but not in muscle or BAT. Fat tissue accounts for a large proportion of body mass (15–30% in healthy subjects and more than 40% in obese subjects) [54]. Moreover, the evidence that reduced gene expression of AMPKα, PGC1α and PPARα was associated with decreased oxidative capacity in WAT [55] and that chronic activation of adipocyte AMP-activated protein kinase (AMPK) increased energy dissipation through enhancing fatty acid oxidation in adipose tissue [56], supports the hypothesis that metabolic change in WAT can contribute to energy expenditure. Nevertheless, further studies are needed to explain how evogliptin up-regulates *Ppargc1a* expression in adipose tissue and whether evogliptin affects PGC1α activation state.

PGC-1α regulates thermogenesis and mitochondrial biogenesis through increasing UCPs expression and augmenting mitochondrial DNA replication in adipocytes [57]. Unexpectedly, evogliptin caused a significant decrease of *Ucp1* expression in WAT, but this result was partly in line with the unaffected rectal temperature in the evogliptin-treated mice. On the other hand, one of mitochondrial components, *Cox4i1* expression, which correlates with increased *Ppargc1a* expression and was not seen with exenatide, was increased by evogliptin treatment.

The 2-week evogliptin treatment significantly ameliorated adipocyte size distribution. Adipocyte size can reflect the metabolic function of adipocytes [58]. It is yet unknown which absolute range of adipocytes sizes is metabolically harmful and no feasible method exists to directly compare adipocyte size from different studies due to discrepancies in sample preparation methods [59]. However, larger adipocytes are known to be metabolically more harmful than smaller ones [58]. In our data, the area of greater than 99% of the adipocytes from lean mice were <2,400 μm^2^. Interestingly, evogliptin treatment significantly reduced the portion of large adipocytes (>3 600 μm^2^), implying the metabolic improvement.

Our study has some limitations. The maximum fat-loss effect of evogliptin has not yet been verified. We previously reported the weight-loss effect of cannabinoid (CB) 1 receptor antagonist (SR 141716A also known as rimonabant which had an approval for anti-obesity drug) in a similar mice model [60]. Rimonabant achieved maximum efficacy 2 to 3 weeks post-dose according to the doses tested. However, the appropriate time for maximum efficacy can differ based on the mode of action involved in the fat-loss effects. We speculate that the fat-loss effect of evogliptin along with improvement in glucose and lipid metabolism, could be amplified with longer-term treatment. Additionally, we used a high-fat diet containing relatively lower carbohydrate, supplying 60% calories from fat. In mice fed on a high-fat diet with lower caloric contribution from fat (45%) with increased sucrose, the contribution of glucose metabolism to fat storage may be more significant.

In conclusion, our findings suggest the beneficial effects of evogliptin at a clinically relevant dose, on whole body composition, particularly whole body fat loss in an established obese mice model. This study verified fat loss by DPP4 inhibition which is likely mediated by increased energy expenditure and alteration in white adipose tissue metabolism. Our findings provide new insight into the role of DPP4 inhibitors in adiposity.

## Supporting Information

S1 Fig Effects on glucose metabolism after 2-week treatment. (PDF)

S2 Fig Body composition changes in Study 3. (PDF)

S3 Fig Analysis of treatment effects on total energy expenditure according to the body composition. (PDF)

S4 Fig Effect on rectal temperature. (PDF)

S5 Fig Effects on other cytokine levels. (PDF)

S6 Fig Effects on gene expression in white adipose tissue after 2-week treatment in HF-DIO mice. (PDF)

S7 Fig Effects of on adipogenesis and PPARα/γ activation. (PDF)

S1 Table Primers and probe for mouse genes used. (PDF)

S2 Table Body composition changes in Study 2 after 2-week treatment. (PDF)

## Abbreviations

AMC: 7-amido-4-methylcoumarin
AMPK: AMP-activated protein kinase
ANCOVA: analysis of covariance
ANOVA: analysis of variance
AUC: the area under the time-blood glucose levels curve
BAT: brown adipose tissue
COX4 (*Cox4il*): cytochrome c oxidase subunit 4 isoform 1
DPP4 (*Dpp4*): dipeptidyl peptidase 4
GLP-1: glucagon-like peptide-1
EE: energy expenditure
HIF-1α: hypoxia-inducible factor -1α
HF: high-fat diet
HF-DIO: high-fat diet-induced obese
PPARγ2: peroxisome proliferator-activated γ2
PGC1α (*Ppargc1a*): peroxisome proliferator-activated receptor-γ coactivator
Rq: respiratory quotient
SREBP1c (*Srebp1c*): sterol response element-binding protein 1c
UCP1 (*Ucp1*): uncoupling protein 1
Rsp3: ribosomal protein S3
VCO_2_: carbon dioxide production VO_2_, oxygen consumption
WAT: white adipose tissue
