# Supplementary material for: Beneficial Effects of Evogliptin, a Novel Dipeptidyl Peptidase 4 Inhibitor, on Adiposity with Increased Ppargc1a in White Adipose Tissue in Obese Mice
Source: PLoS One. 2015 Dec 3;10(12):e0144064. doi: 10.1371/journal.pone.0144064 (PMC4669177; doi:10.1371/journal.pone.0144064)
Supplement: S1 Fig — (PDF) [file pone.0144064.s001.pdf]

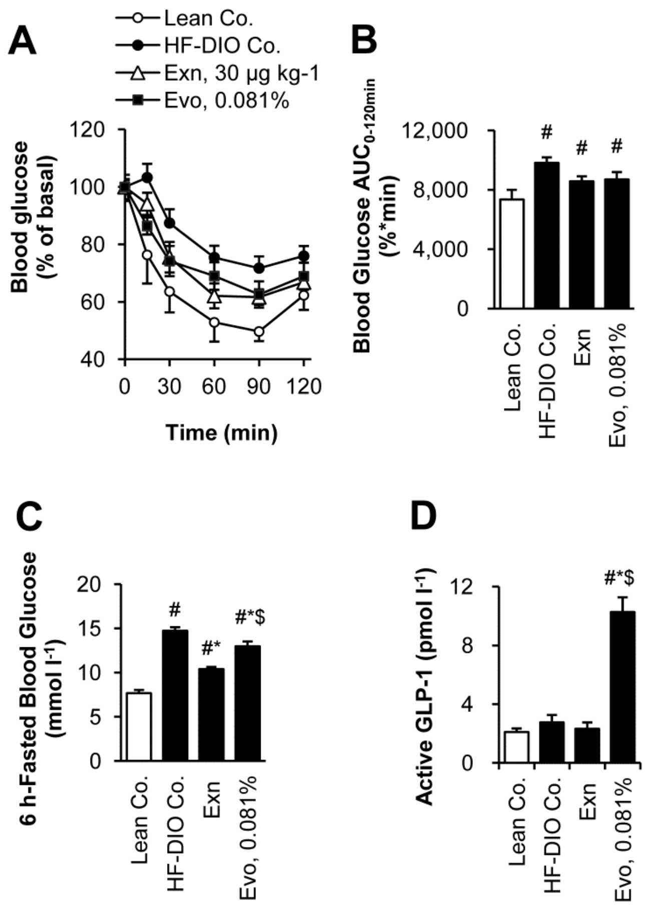

S1 Fig. Effects on glucose metabolism after 2-week treatment (ipITT). HF-DIO mice were allocated into three groups according to body weight and fed blood glucose. After 2-week treatment of 30 εg kg^{-1} exenatide or 0.081% (w/w) evogliptin, insulin tolerance was tested in 6 h-fasted mice. Twenty-four hours after the last dosing, blood glucose levels were measured at the indicated time points for 120 min following i.p. injection of insulin (0.75 units/kg; Sigma, I9278). **(A)** Blood glucose levels were denoted as percentages to the baseline glucose levels. **(B)** The area under the time-blood glucose curve was computed. Exenatide and evogliptin tended to reduce 13% (P=0.15) and 11% (P=0.093) of glucose AUC compared to HF-DIO control, respectively. **(C)** When 6 h-fasted blood glucose levels right before the insulin injection were compared between treatments, exenatide and evogliptin showed significant reductions of 29.5% and 11.9% compared to the HF-DIO control, respectively. **(D)** At the end of insulin tolerance test, plasma was collected in heparinized tubes containing sitagliptin to minimize the GLP-1 degradation. Evogliptin, but not exenatide, increased biologically active GLP-1 (3.7 folds vs. HF-DIO control). Exenatide treatment did not significantly alter active GLP-1 levels (-16%, P=0.631). n=8 animals/group; #, $P < 0.05$ vs. Lean Co.; *, $P < 0.05$ vs. HF-DIO Co.; $, $P < 0.05$ vs. exenatide by One-way ANOVA
