# Supplementary material for: Beneficial Effects of Evogliptin, a Novel Dipeptidyl Peptidase 4 Inhibitor, on Adiposity with Increased Ppargc1a in White Adipose Tissue in Obese Mice
Source: PLoS One. 2015 Dec 3;10(12):e0144064. doi: 10.1371/journal.pone.0144064 (PMC4669177; doi:10.1371/journal.pone.0144064)
Supplement: S2 Fig — (PDF) [file pone.0144064.s002.pdf]

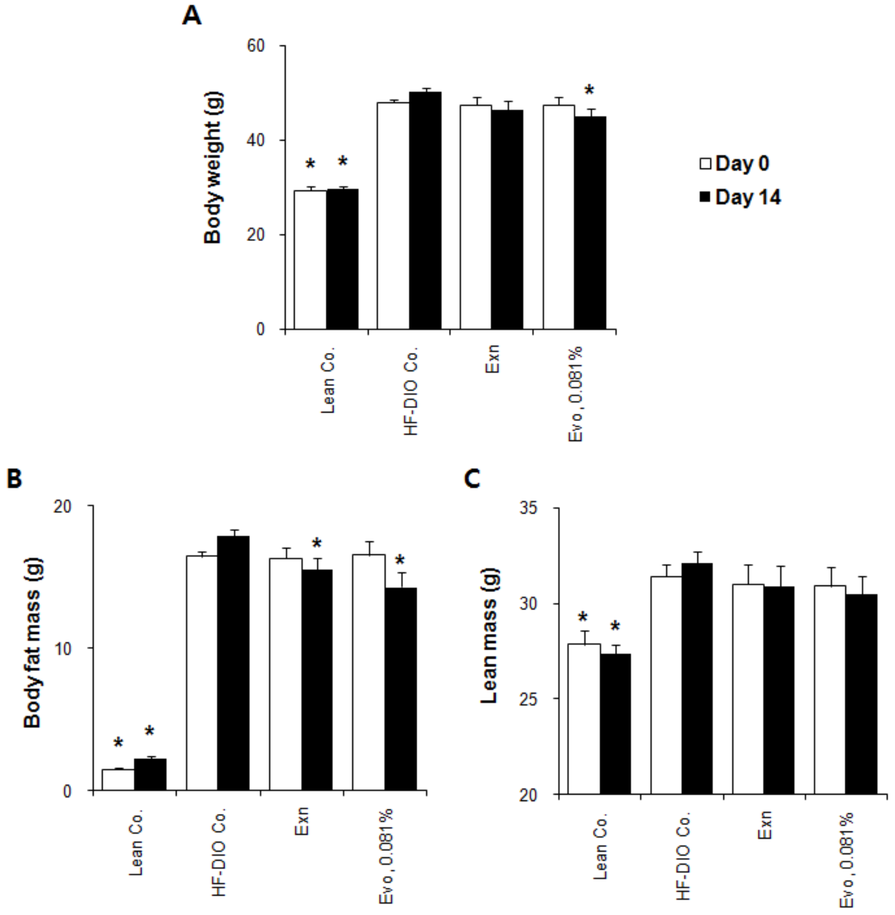

S2 Fig. Body composition changes in Study 3. After 2-week treatment of 30 əg kg^{-1} exenatide or 0.081% evogliptin [Study 3 as described in *Method* section], **(A)** body weight, **(B)** whole body fat mass, and **(C)** lean mass were assessed before (Ã) and after 2-week treatment (Â) in HF-DIO mice (n=8/group). *, $P < 0.05$ vs. HF-DIO control by One-way ANOVA at each time point.
