# Supplementary material for: Beneficial Effects of Evogliptin, a Novel Dipeptidyl Peptidase 4 Inhibitor, on Adiposity with Increased Ppargc1a in White Adipose Tissue in Obese Mice
Source: PLoS One. 2015 Dec 3;10(12):e0144064. doi: 10.1371/journal.pone.0144064 (PMC4669177; doi:10.1371/journal.pone.0144064)
Supplement: S3 Fig — (PDF) [file pone.0144064.s003.pdf]

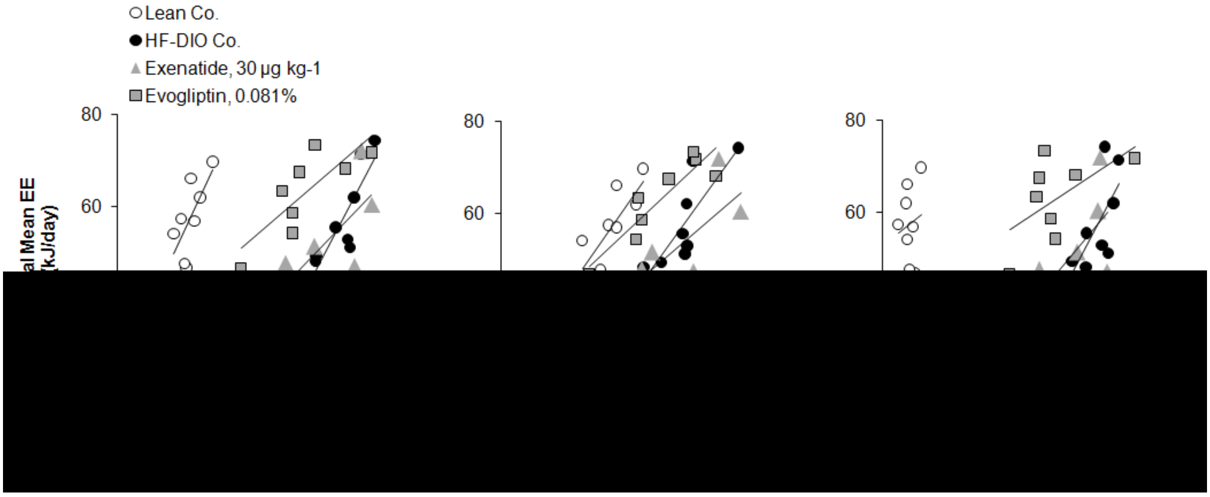

[redacted]

S3 Fig. Analysis of treatment effects on total energy expenditure according to the body composition. According to the previous report (Tschöp MH, et al., *Nat Methods.* 2011; 9: 57-63), energy expenditure was plotted vs. lean mass or fat mass in addition to body weight. Solid lines show fitted regression (n=8/group).
