# Supplementary material for: Beneficial Effects of Evogliptin, a Novel Dipeptidyl Peptidase 4 Inhibitor, on Adiposity with Increased Ppargc1a in White Adipose Tissue in Obese Mice
Source: PLoS One. 2015 Dec 3;10(12):e0144064. doi: 10.1371/journal.pone.0144064 (PMC4669177; doi:10.1371/journal.pone.0144064)
Supplement: S1 Table — (PDF) [file pone.0144064.s008.pdf]

S1 Table. Primers and probe for mouse genes used

Gene name (Accession No.)	*Primers*	*TaqMan Probe*
Ppargc1a (#19017)	F: 5'-AGAAGCGGGAGTCTGAAAGG-3'<br>R: 5'-CAGTTCTGTCCGCGTTGTG-3'	-
Cox4i1 (#12857)	F: 5'-TGCTAGCCGCAGGCATTACT-3'<br>R: 5'-TCTGGGTGCCCAAAGAATCA-3'	5'-6FAM-CGCTGGAGGAGGGGACCCAATTCTCTA-TAMRA-3'[a]
Pparg2 (#19016)	F: 5′-GGTGAAACTCTGGGAGATTC-3′<br>R: 5′-CAACCATTGGGTCAGCTCTT-3′	-
Dpp4 (#13482)	F: 5'-GTGGCAAGAGGGGATCACTA-3′<br>R: 5'-CCCAGCCTGTGGTACTCATT-3′	-
Hif1a *(#15251)*	F: 5'-TCAAGTCAGCAACGTGGAAG-3'<br>R: 5'-TATCGAGGCTGTGTCGACTG-3'	
Srebp1c (#20787)	F: 5′-CCAGCAGGTCCCAGTTGTAT-3′<br>R: 5′-AGGTACTGTGGCCAAGATGG-3′	-
Rps3 (#27050)	F: 5'-ATCAGAGAGTTGACCGCAGTTG-3'<br>R: 5'-AATGAACCGAAGCACACCATAG-3'	-
Ucp1	#22227: P/N4331182, commercialized ABI gene expression probe and primer set	

a. Strum JC, Shehee R, Virley D, Richardson J, Mattie M, Selley P, et al. Rosiglitazone induces mitochondrial biogenesis in mouse brain. J Alzheimers Dis. 2007; 11: 45-51.
b. Abbreviations: ***Ppargc1a*** for peroxisome proliferator-activated receptor-γ coactivator 1α, ***Cox4il*** for mitochondrial complex IV subunit cytochrome C oxidase subunit 4, ***Pparg2*** for peroxisome proliferator-activated receptor-γ2, ***Dpp4*** for dipeptidyl peptidase 4, ***Hif1a*** for hypoxia inducible factor 1- α, ***Srebp1c*** for sterol regulatory element binding protein-1c, and ***Rps3*** for ribosomal protein S3
