# Supplementary material for: Beneficial Effects of Evogliptin, a Novel Dipeptidyl Peptidase 4 Inhibitor, on Adiposity with Increased Ppargc1a in White Adipose Tissue in Obese Mice
Source: PLoS One. 2015 Dec 3;10(12):e0144064. doi: 10.1371/journal.pone.0144064 (PMC4669177; doi:10.1371/journal.pone.0144064)
Supplement: S2 Table — (PDF) [file pone.0144064.s009.pdf]

S2 Table. Body composition changes in Study 2 after 2-week treatment

Treatment groups		Body wt (g)		Fat mass (g)		Lean mass (g)	
		Day 14	Changes vs. Day 0	Day 14	Changes vs. Day 0	Day 14	Changes vs. Day 0
**Lean Co.**		30.4±0.29	*+0.36*[c]	2.25±0.12	*+0.30*[c]	28.2±0.38	*+0.06*
**HF-DIO Co.**		37.9±0.71[a]	*+0.23*	10.56±0.63[a]	*+0.62*	27.3±0.40	*-0.40*
**Exn**		36.0±1.11[a]	*-1.51*	9.19±0.60[a]	*-0.74*	26.9±0.67	*-0.77*
**Evogliptin**	**0.027%**	37.1±1.32[a]	*-1.47*	9.43±0.66[a]	*-0.71*	27.6±0.80	*-0.77*
	**0.081%**	34.9±0.77[a]	*-2.88*	7.75±0.45[ab]	*-2.26*	27.2±0.65	*-0.62*
	**0.27%**	32.8±0.91[b]	*-4.74*[c]	6.49±0.35[ab]	*-3.59*[c]	26.3±0.68	*-1.15*

8 animals/group; a, $P < 0.05$ vs. Lean Co. and b, $P < 0.05$ vs. HF-DIO Co. by one-way ANOVA; c, $P < 0.05$ vs. HF-DIO Co. by RM two-way ANOVA
